# Remote consultations in London – a comparison between mental health and cardiology pathways

**DOI:** 10.1192/j.eurpsy.2023.200

**Published:** 2023-07-19

**Authors:** M. Pinto da Costa, NIHR ARCs (Chris Chaloner, Nirandeep Rehill, Kristoffer Halvorsud, Rosalind Raine, Fiona Stevenson, Gabriele Kerr, Thomas Beaney, Geva Greenfield, Benedict Hayhoe, Ceire Costelloe, Massar Dabbous, Gideon Gideon, Eoin Gogarty, Fiona Gaughran)

**Affiliations:** Institute of Psychiatry, Psychology & Neuroscience, King’s College London, London, United Kingdom

## Abstract

**Abstract:**

As a response to the COVID-19 pandemic, in March 2020 a shift to remote consulting for routine primary care and outpatient appointments was instructed. With the aim to inform whether and how remote consultations should continue post-pandemic, the London NIHR Applied Research Collaborations (ARCs) conducted a research-grade evaluation of the impact of this shift in London, focusing on cardiology and mental health as exemplar pathways.

Quantitative methodology aimed to explore patterns of healthcare use, efficiency, and clinical outcomes using de-identified patient-level health records datasets in North-West London and South London.

Qualitative interviews with clinicians and patients with experience in remote consultations sought to develop a more in-depth understanding of the experience of the move to remote consulting.

**Disclosure of Interest:**

None Declared

